# Osteoblastic differentiation of bone marrow mesenchymal stromal cells in Bruck Syndrome

**DOI:** 10.1186/s12881-016-0301-7

**Published:** 2016-05-04

**Authors:** Carla M. Kaneto, Patrícia S. P. Lima, Dalila Lucíola Zanette, Thiago Yukio Kikuchi Oliveira, Francisco de Assis Pereira, Julio Cesar Cetrulo Lorenzi, Jane Lima dos Santos, Karen L. Prata, João M. Pina Neto, Francisco J. A. de Paula, Wilson A. Silva

**Affiliations:** Department of Genetics, Medical School of Ribeirão Preto, Universidade de São Paulo, Ribeirão Preto, São Paulo Brazil; Regional Blood Center of Ribeirão Preto and National Institute of Science and Technology in Cell Therapy, Ribeirão Preto, Brazil; Department of Natural Science, Universidade Estadual do Sudoeste da Bahia, Vitória da Conquista, Bahia Brazil; Department of Clinical Medicine, Faculdade de Medicina de Ribeirão Preto, Universidade de São Paulo, Ribeirão Preto, São Paulo Brazil; Department of Biological Science, Universidade Estadual de Santa Cruz, Ilheus, BA Brazil; Laboratory of Molecular Immunology, The Rockefeller University, New York, NY USA; Department of Internal Medicine and Pathology, Universidade Federal de Sergipe, Aracaju, SE Brazil

**Keywords:** Bruck syndrome, Osteogenesis Imperfecta, Bone marrow mesenchymal stromal cell, Osteogenic differentiation, Gene expression

## Abstract

**Background:**

Osteogenesis Imperfecta (OI) (OMIM %259450) is a heterogeneous group of inherited disorders characterized by increased bone fragility, with clinical severity ranging from mild to lethal. The majority of OI cases are caused by mutations in *COL1A1* or *COL1A2*. Bruck Syndrome (BS) is a further recessively-inherited OI-like phenotype in which bone fragility is associated with the unusual finding of pterygia and contractures of the large joints. Notably, several studies have failed to show any abnormalities in the biosynthesis of collagen 1 in BS patientes. Evidence was obtained for a specific defect of the procollagen telopeptide lysine hydroxylation in BS, whereas mutations in the gene *PLOD2* have been identified. Recently, several studies described FKBP10 mutations in OI-like and BS patients, suggesting that *FKBP10* is a bonafide BS locus.

**Methods:**

We analyzed the coding region and intron/exon boundaries of *COL1A1*, *COL1A2, PLOD2* and *FKBP10* genes by sequence analysis using an ABI PRISM 3130 automated sequencer and Big Dye Terminator Sequencing protocol. Mononuclear cells obtained from the bone marrow of BS, OI patients and healthy donors were cultured and osteogenic differentiation was induced. The gene expression of osteoblast specific markers were also evaluated during the osteoblastic differentiation of mesenchymal stem cell (MSC) by qRT-PCR using an ABI7500 Sequence Detection System.

**Results:**

No mutations in *COL1A1*, *COL1A2* or *PLOD2* were found in BS patient. We found a homozygous 1-base-pair duplication (c.831dupC) that is predicted to produce a translational frameshift mutation and a premature protein truncation 17 aminoacids downstream (p.Gly278ArgfsX95). The gene expression of osteoblast specific markers *BGLAP*, *COL1A1*, *MSX2*, *SPARC* and *VDR* was evaluated by Real Time RT-PCR during differentiation into osteoblasts and results showed similar patterns of osteoblast markers expression in BS and healthy controls. On the other hand, when compared with OI patients, the expression pattern of these genes was found to be different.

**Conclusions:**

Our work suggests that the gene expression profiles observed during mesenchymal stromal cell differentiation into osteoblast are distinct in BS patients as compared to OI patients. The present study shows for the first time that genes involved in osteogenesis are differentially expressed in BS and OI patients.

**Electronic supplementary material:**

The online version of this article (doi:10.1186/s12881-016-0301-7) contains supplementary material, which is available to authorized users.

## Background

Bruck Syndrome (OMIM %259450) is a rare disorder in which joint contractures are associated with bone fragility and, because of this OI-like phenotype, the diagnosis of Osteogenesis Imperfecta (OI) is subsequently considered. Patients with BS usually exhibit multiple joint contractures and pterygia, as observed in arthrogryposis multiplex congenita. In the subsequent weeks, multiple fractures of the ribs and long bones are detected, as well as a diaphyseal bending, kyphoscoliosis, and persistent wormian bone in the calvarium; these features are strongly reminiscent of OI [1, 2]. In contrast to OI, these patients do not have blue sclera or hearing loss. Mental development is also normal and reports suggest an autosomal recessive inheritance pattern, but only approximately 27 cases have been reported in the literature [3]. Notably, several studies have failed to show any abnormalities in the biosynthesis of collagen 1 and 2 as it can be noted in OI individuals [4], but recent reports have shown that mutations in other genes, like *PLOD2* and *FKPB10,* can result in both BS and Autosomal-Recessive OI (AR-OI) phenotypes with variable degrees of bone fragility and joint contractures [5], suggesting the necessity for reassessing the current classification of OI.

A specific defect of procollagen telopeptide lysine hydroxylation in BS and mutations in the gene *PLOD2* have been identified (BS type 2); yet, the clinical and radiographic features of *PLOD2*-associated BS have not been described in detail, and only a large family study showed evidence of linkage to chromosome 17 (designated BS type 1) [6]. Hydroxylysine residues have some important functions in collagens, such as the role in stabilization of the intermolecular collagen cross-links, which pattern has been found to differ from tissue to tissue [7]. It has been shown that the amount of hydroxyallysine-derived cross-links is related to the irreversible accumulation of collagen in fibrotic tissues whereas the inhibition of these crosslinks results in the formation of a collagen that is easier to degrade and can thereby prevent unwanted collagen accumulation [8]. An important step in this process is the *cis-trans* isomerization of peptide bonds. This process can be catalyzed by peptidylprolyl *cistrans*-isomerases (cyclophilins and FKBPs), like FKBP65 [9].

Novel mutations in *FKBP10*, which encodes FKBP65, have been reported to cause a recessive OI-like phenotype. In a study of a cohort of consanguineous Turkish families with moderately severe inherited OI, Alanay et al. (2010) found mutations in *FKBP10* and determined that *FKBP10* mutations affect type I procollagen secretion, identifying a previously unrecognized mechanism in the pathogenesis of OI [10]. Kelley et al. (2011) also described *FKBP10* mutations in five families with OI-like bone fragility in association with congenital contractures [11]. These studies, combined with other published works, confirm that *FKBP10* is a bonafide BS locus [3, 12, 13].

Despite this, more research into BS and OI phenotypic heterogeneity are necessary and there is little information about gene expression in undifferentiated and differentiated bone marrow stromal cells (BMSCs) from these patients. Uzawa et al. (1999) showed that normal undifferentiated BMSCs have low *PLOD2* expression levels, whereas fully osteoblast differentiated BMSCs exhibited a significantly elevated level of *PLOD2* mRNA, suggesting an association between *PLOD2* expression and the tissue-specific collagen cross-linking pattern, which is important in the onset of matrix mineralization [14].

Despite this, there is no information concerning *PLOD2* and other genes expressed in BMSCs from BS patients. Our work provides information about the gene expression of undifferentiated and osteoblast differentiated BMSCs from a BS patient compared with OI and health controls. We have monitored the surface marker profile and the differentiation potential of these cells during their in vitro expansion. Finally, we have analyzed osteoblast specific markers gene expression levels during osteogenic differentiation. BS cells were also screened for *COL1A1*, *COL1A2*, *PLOD2* and *FKBP10* mutations. Results showed that, when compared with OI patients, the expression pattern of osteoblastic specific genes was found to be different.

## Methods

### Clinical report

The patient - a boy- was the first child of non-consanguineous parents born at term by caesarian section in 2005 after an uneventful pregnancy. The patient’s weight was 2910 g (Z-score = −1.7 SD) and his height was 43 cm (Z-score = −4.0 SD). Fractures of the left arm and left clavicle were diagnosed immediately after birth. During the first 4 months, the boy had additional fractures of the ribs, lumbar spine (L1), and left and right legs. At the age of 6 months, his weight was 5985 g and he was admitted for the first time at the Medical School of Ribeirão Preto Hospital, University of São Paulo. The boy had blue-gray sclera and pterygia were present on both elbows and knees, which had severe limited extension. He also showed contractures at the wrists, bowed legs and bilateral clubfeet. Radiographic exams of the long bones and skull revealed diffuse osteopenia. Serum levels of calcium, phosphate and parathyroid hormone determined at the age of 7 months were considered normal. Thus, after the parents signed an informed consent, the patient received the first dose of disodium pamidronate, which was maintained over a period of 5 years. Cyclical intravenous disodium pamidronate was given as previously suggested by Glorieux and his group [15]. Up to the age of 2 years, pamidronate was administered over a period of 1 h by intravenous (i.v.) infusion at the dose of 0.5 mg/kg for 3 days every 2 months. Up to the age of 3 years, the patient was treated with pamidronate over a period of 1 h i.v. at the dose of 0.75 mg/kg, for 3 days, every 3 months. The pamidronate dosage was thereafter adjusted to 1 mg/kg, for 3 days, every 4 months. Only two new fractures occurred throughout this period and the patient was then able to sit up independently. Pterygia persisted at both elbows and knees. His mental development was normal. The treatment was well tolerated and no side-effects were observed, except fever after administration of the first dose. The patient became more active and less agitated. This study was carried out with the approval of the Medical Ethics Committee of the HC-FMRP-USP (No. 10188/2007), and an informed consent was obtained from all the participants.

### In vitro expansion of human bone marrow-derived stromal cells

Mononuclear cell fractions (MNC) were derived from bone marrow of four OI patients (aged 15–39 years (25,75 ± 9,97)), two normal donors (23 and 24 years), and one BS patient (6 year old), who gave consent after full information and approval by the Medical Ethics Committee of the HC-FMRP-USP. All MNC were isolated from bone marrow aspirates at iliac crest. MNC were seeded at a density of 1 × 10^5^ per cm^2^ and were cultured for 24 h. After this period, non-adherent cells were removed by medium exchange. The adherent cells were cultured in an expansion medium containing alpha-minimum essential medium (Gibco), 15 % FBS (Gibco), 100U/mL penincillin, 100 μg/ML streptomycin, and 2 mM L-glutamine under a humidified atmosphere of 5 % CO_2_ at 37 °C. The medium was replaced three times a week. After reaching a confluence of 75–85 %, cells were detached with 0.05 % trypsin and were replated at a density of 5 × 10^5^ cells. Cells were expanded until the end of the third passage, when osteogenic differentiation was induced with an expansion medium supplemented with 0.01 mM dexamethasone, 200 μM ascorbic-acid-2-phosphate and 10 mM β–glycerophosphate. RNA was harvested at seven time points during the osteogenic differentiation period (D0, D + 1, D + 2, D + 7, D + 12, D + 17 and D + 21).

### In vitro differentiation of human bone marrow-derived MSC in osteoblasts and adipocytes

For the induction of osteogenesis, MSC were seeded at a density of 10^4^ cells/cm^2^ in an expansion medium. After 24 h, the differentiation was induced by medium exchange. The osteogenic differentiation medium was based on the expansion medium supplemented with 0.01 mM dexamethasone, 200 μM ascorbic-acid-2-phosphate and 10 mM β–glycerophosphate. In addition to the expansion medium, the adipogenic differentiation medium contained 0.1 μM dexamethasone, 50 μM indomethacin, and 5 μg/mL insulin. The media were changed three times a week for 21 days. Osteogenic differentiation was detected by Von Kossa staining of mineralized matrix on day 14, whereas adipogenic differentiation was detected on day 10 by a Sudan II assay that stains adipocyte-specific fat vacuoles.

The Von Kossa assay was performed with silver nitrate. The medium was removed and the cultures were washed with PBS, fixed with 4 % paraformaldehyde for 15 min, washed again with distilled water, and incubated with 5 % silver nitrate for 30 min. After incubation, the staining solution was removed and the cultures were washed with 5 % sodium thiosulfate. Next, the cells were stained with Harris hematoxylin for 5 min. Finally, cells were washed with distilled water so as to remove excess color.

For Sudan II staining, a 2 % Sudan stock solution in ethanol was prepared. The medium was removed; the cultures were washed with PBS, fixed with 4 % paraformaldehyde for 15 min, washed again with distilled water, and incubated with Scarlet Sudan for 5 min. After incubation, the staining solution was removed and the cultures were washed with 70 % ethanol. Then, the cells were stained with Harris hematoxylin for 5 min. Finally, the cells were washed with distilled water for removal of excess color. Cells were analyzed with an Axioscope 2.0 Zeiss microscope equipped with an AxioCam HR camera (Carl Zeiss, Germany). The cells were harvested and analyzed at 14 and 21 days for osteogenic and adipogenic differentiation, respectively.

### Surface marker profiling by flow cytometry analysis

For flow cytometry analysis, the cells were detached with 0.05 % trypsin and were washed with PBS. A volume of 1 x 10^5^ cells per tube were stained for 15 min and were afterwards incubated in an ice bath in the dark with direct PE or FITC conjugated mouse anti-human monoclonal antibodies (Becton Dickinson) recognizing CD45, CD14, CD29, CD105, CD90, CD34, CD31, and CD73. After antibody incubation, the cells were washed with PBS/sodium azide and were resuspended in PBS in the volume required for analysis. Immunoglobulin isotype incubation was carried out as a negative control. FACS (*Fluorescence Activated Cell Sorting*) analysis was performed with FACSort™ (Becton Dickinson).

### RNA and DNA purification

Total RNA was isolated with TRIzol reagent (Invitrogen) and concentration and quality were assessed with Nanodrop 2000 spectrophotometer (Thermo Fisher Scientific, Waltham, MA, USA). The High Capacity cDNA Reverse Transcription Kit (Applied Biosystems) was used to synthesize cDNA of 1 μg RNA, following manufacturer’s recommendations.

Genomic DNA was obtained from undifferentiated BMSCs in the third passage, using the Wizard Genomic DNA Purification Kit (Promega) according to the manufacturer’s instructions. DNA concentration was assessed with Nanodrop 2000 spectrophotometer (Thermo Fisher Scientific, Waltham, MA, USA) and quality was analyzed by electrophoresis on 2 % agarose gel. For PCR reactions, DNA was diluted to 100 ηg/μL.

### Mutation analysis

DNA sequencing of PCR-amplified *COL1A1*, *COL1A2*, *PLOD2* and *FKBP10* gene fragments covering the entire coding region and intron/exon boundaries was carried out using an ABI PRISM 3130 automated sequencer and Big Dye Terminator Sequencing protocol. All primer sequences and the respective annealing temperatures used for PCR amplification of *COL1A1, COL1A2*, *PLOD2* and *FKBP10* are available as an Additional file 1. PCR reactions were performed with a total volume of 25 uL ((NH_4_)_2_SO_4_ 2 M, Tris–HCl 2 M, MgCl_2_ 1 M and 1 % Tween 20 Buffer, 2.5 μM of each primer at 5 pmol/uL, 1.5 mM dNTP, 1U Taq DNA Polymerase (Biotools) and 4 uL of DNA at 5 ng/uL). The amplification parameters were: 94 °C for 6 min, 35 cycles of 94 °C for 1 min, annealing temperature for 1 min and 72 °C for 1 min. The founded mutations were confirmed in a second sample through another PCR reaction with the use of Platinum® Taq DNA Polymerase High Fidelity (Invitrogen, São Paulo, Brazil), followed by sequencing.

### Expression analysis

RT-PCR assays were performed to detect the expression levels of osteoblast specific marker genes *BGLAP* (osteocalcin), *COL1A1* (collagen 1A1), *MSX2* (homeobox gene MSX2), *SPARC* (osteonectin), and *VDR* (vitamin D receptor) to prove the osteoblast phenotype. All primer sequences used for PCR amplification are available as an Additional file 1. qRT-PCR amplification mixtures contained 20 ng template cDNA, 2X Power SYBR Green Master mix buffer (10 μL) (Applied Biosystems) and 400 to 600 nM forward and reverse primers in a final volume of 20 μL. All reactions were duplicated using an ABI7500 Sequence Detection System (Applied Biosystems, Foster City, CA, USA), under the following conditions: 95 °C, for 10 min, followed by 40 cycles, at 95 °C, for 15 s, and 60 °C, for 1 min. Total RNA input was normalized based on the Ct values obtained for the housekeeping gene *GAPDH*. Experiments with coefficients of variation greater than 5 % were excluded. For all reactions, each run was completed with a melting curve analysis to confirm the specificity of amplification and lack of primer dimers. Reactions were run in triplicates and a no-template control (NTC) and no-reverse transcription controls (No-RT) were also included. The relative quantification of gene expression was carried out using the mathematical model described in Pfaffl et al. (2001). Gene expression was analyzed using 1-way analysis of variance, followed by a Kruskal-Wallis test with Prism 3.0 (GraphPad Software, San Diego, CA, USA). All differences were considered significant at values of *P* < 0.05.

## Results

### BS bone marrow-derived stromal cells and normal cells have similar surface marker profiles and in vitro differentiation potential

**Fig. 1:**
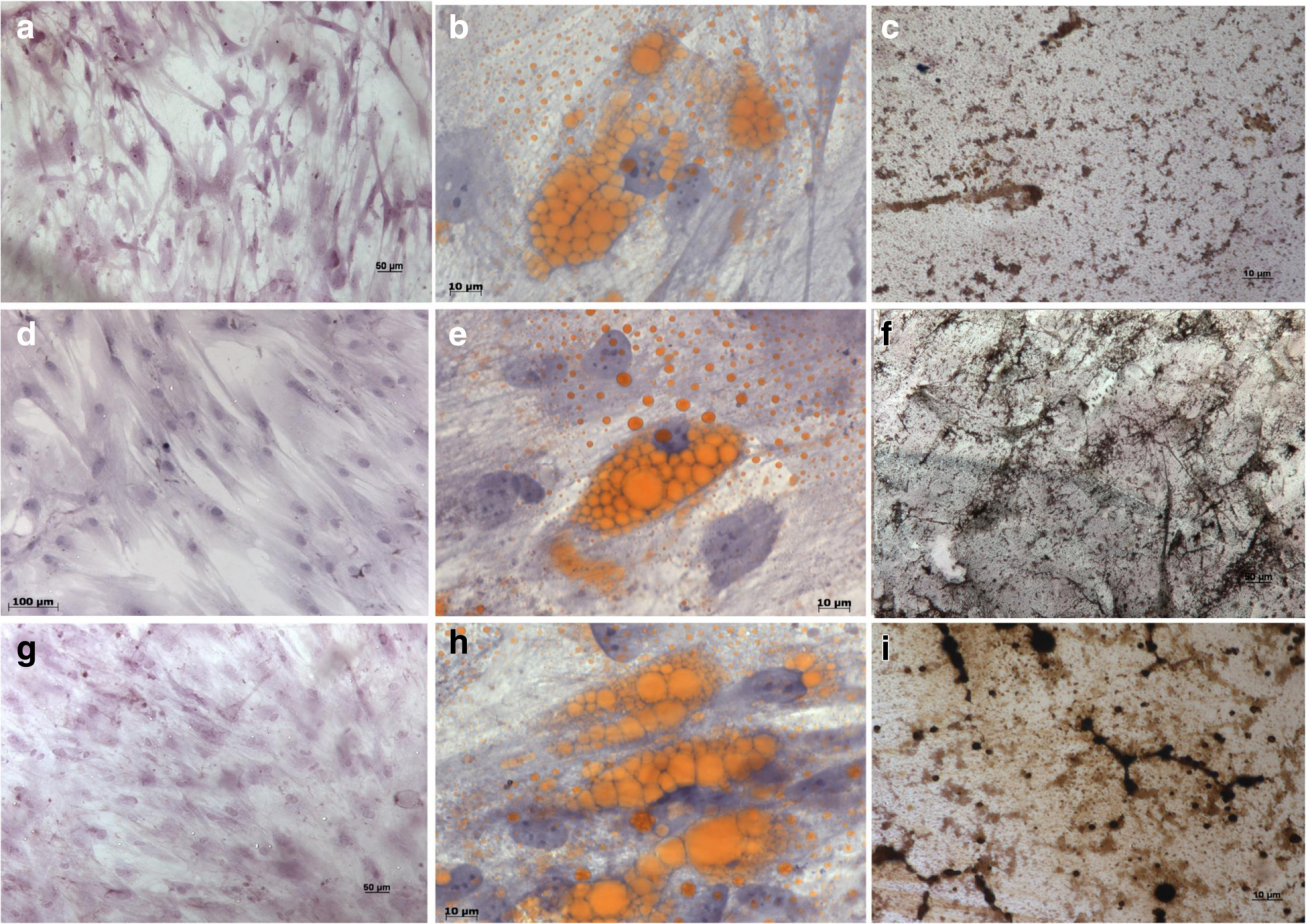
Adipogenic and Osteogenic differentiation of mesenchymal stem cells (MSCs) after passage 3. **a**. BS patient; not induced MSC; undifferentiated, magnitude order: 630X. **b**. BS cells differentiated into adipocyte after staining with Sudan II-Scarlat assay, magnitude order: 630X **c**. BS cells differentiated in osteoblast after staining with Von Kossa assay; magnitude order: 10X. **d** normal donor; not induced MSC; magnitude order: 100X. **e**. Normal donor cells differentiated into adipocyte, magnitude order: 630X. Sudan II staining showed MSC adipogenic differentiation after 10 days. **f**. normal donor cells differentiated in osteoblast; magnitude order: 10X. **g**. OI patient, undifferentiated MSC; magnitude order: : 630X. **h**. OI patient cells differentiated into adipocyte; magnitude order: 630X **i**. OI patients’ cells differentiated in osteoblast; magnitude order: 10X. Von Kossa staining showed mineralization nodules (black) and MSCs morphology changed from spindle to cuboid shape after 14 days

In order to evaluate patients BMSCs population, we verify that MSCs derived from BS patient have similar levels of CD45-, CD14-, CD34-, CD31-, CD29+, CD13+, CD90+, CD73+, and CD105+ surface markers compared with OI and healthy donors MSCs (Additional file 2). Results show that BS, OI, and normal donor BMSCs depicted homogenous cell population, demonstrating that MSC did not alter their physical and morphological properties in response to the syndrome. BS derived cells also showed regular osteogenic and adipogenic differentiation abilities (Fig. 1).

### Mutation analysis

All OI patients showed the characterized mutations in *COL1A1* or *COL1A2* genes previously described [16]. In *COL1A1*: 1 missense mutation (p. Gly290Glu) have been identified in patient MOOI3, 1 nonsense mutation (Arg1026Ter) have been identified in patient MOOI1 and 1 out-of-frame insertion mutation (p. Leu69GlufsX74) have been identified in patient MOOI4. In *COL1A2*: 1 missense mutation (p. Gly835Ser) have been identified in patient MOOI2. Those mutations lead to amino acid substitutions that are probably responsible for the observed phenotype. We also found two *COL1A1* and one *COL1A2* variations in BS patient that were considered as non-pathogenic, as they do not alter amino acids [16].

**Fig. 2:**
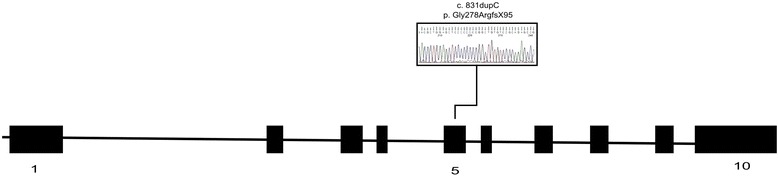
Schematic view of *FKBP10* coding region showing a homozygous C duplication at position 831

In BS patient, no mutations were found in *PLOD2* gene. All 10 coding exons in the *FKBP10* gene were analyzed by DNA sequencing. We found a homozygous 1-base-pair duplication (c.831dupC) in BS patient, which is the same as reported by Alanay et al. (2010), Kelley et al. (2011) and Caparros-Martin et al. (2013) [10, 11, 17] (Fig. 2). This mutation is predicted to produce a translational frameshift mutation and a premature protein truncation 95 aminoacids downstream (p.Gly278ArgfsX95). NM_021939.3 was the reference sequence used for FKBP10 cDNA analysis and the mutation was confirmed in the heterozygous state in only one of the progenitors from whom DNA was available.

### Expression of osteoblast specific marker genes

Osteogenesis was monitored by microscopy during the entire in vitro differentiation period. The first signs of calcification appeared as black regions within the cell monolayer in approximately ten days. The maximum extracellular matrix calcification was observed after 21 days of differentiation.

**Fig. 3:**
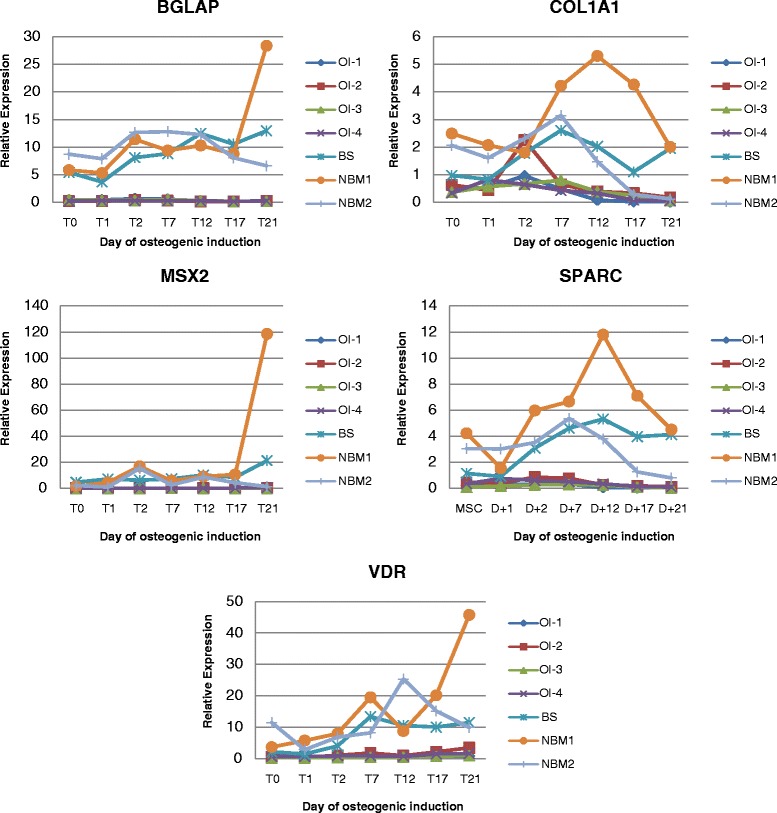
Gene expression of osteoblast markers measured by Real Time RT-PCR at seven different points (MSC, D + 1, D + 2, D + 7, D + 12, D + 17 and D + 21). Legend: OI1-4: Osteogenesis Imperfecta patients; BS: Bruck Syndrome patient; NBM1-2: normal bone marrow donors. Each value represents the mean of two independent experiments

We also observed similar patterns of expression of osteoblast markers: *BGLAP* (osteocalcin), *COL1A1* (collagen 1A1), *MSX2* (homeobox gene MSX2), *SPARC* (osteonectin), and *VDR* (vitamin D receptor) (Fig. 3) between BS and healthy controls osteoblasts. On the other hand, when compared with OI patients, the expression pattern of these genes showed significantly higher expression levels than those observed in OI patients, for which only a slight increase could be detected during in vitro differentiation (Fig. 3).

## Discussion

Bruck syndrome manifests with combined features of arthrogryposis and osteogenesis imperfecta. The main features are osteoporosis, bowing of the long bones, scoliosis due to vertebral deformities, and congenital joint contractures. The presence of arthrogryposis differentiates this syndrome from “classical” Osteogenesis Imperfecta [18], but recent reports have shown that some mutations can result in both BS and Autosomal-Recessive OI (AR-OI) phenotypes with variable degrees of bone fragility and joint contractures [5], calling for the reclassification of OI. BS patients do not exhibit blue sclera or hearing loss and present normal mental development, although the extremely low number of cases ever reported in the literature (approximately 27) limits the knowledge about this condition [3].

The surface marker profile was analyzed by flow cytometry and revealed that BS, OI, and normal donor BMSCs depicted homogenous cell population, demonstrating that MSC did not alter their physical and morphological properties in response to the syndrome; this supports our observation that MSC maintained their undifferentiated phenotype and remained capable of differentiating during ex vivo expansion.

Our study has also shown that BMSCs from BS patients are capable of undergoing osteogenic and adipogenic differentiation when cultivated under appropriate conditions, in the same way as OI patients and healthy control cells. The time necessary for complete differentiation was also similar, suggesting that their capacity for mesenchymal lineage differentiation was not compromised.

Several studies have failed to show any abnormalities in the biosynthesis of *COL1A1* and *COL1A2* from patients with BS, as observed in the majority of OI individuals. In our study, two *COL1A1* and one *COL1A2* synonym mutations were found in the BS patient, but they did not correspond to modifications in the amino acid sequence, suggesting that these mutations are not responsible for the observed phenotype.

Although all three described *PLOD2* mutations associated to BS are located in exon 17, the entire coding region of this gene was directly sequenced and no mutations were found. *PLOD2* mutations are described as a cause for BS type 2, whereas another type 1 - BS - is associated with a linkage to chromosome 17, as described by Bank et al. [1999], who mapped the BS locus of an 18-centimorgan region on chromosome 17p12.

Analyzing *FKBP10* by DNA sequencing, a homozygous 1-base-pair duplication (c.831dupC) was found. This mutation is predicted to produce a translational frameshift mutation and a premature protein truncation (p.Gly278ArgfsX95). This mutation, also described by Alanay et al. (2010), Kelley et al. (2011) and Caparros-Martin et al. (2013), results in a stop codon 17 aminoacids downstream in the third PPIase (peptidylprolyl cis-trans Isomerase) domain of the FKBP65 protein, which is a known chaperone for type I procollagen [9, 19] and that have been implicated the folding of the proline-rich tropoelastin monomer. Alanay et al. (2010) showed aggregated intracellular type I procollagen in FKBP65 mutant cells, suggesting a functional defect in the chaperone activity of the FKBP65 that is consistent among BS patients.

Type-I collagen is a heterotrimeric molecule composed of two α1 chains and one α2 chain that is the predominant matrix molecule in bones. The biosynthesis of collagen molecule is a long and complicated process involving a number of post-translational modifications. The hydroxylation of specific peptidyl lysine (Lys) residues is critical for the glycosylation and cross-linking of the collagen molecule and is performed by lysyl hydroxylases (LH) [14]. Lys hydroxylation in the nontryple helical domains of the collagen molecule is particularly important in determining the cross-link patterns that are known to differ from one tissue to another. Furthermore, several studies have demonstrated the occurrence of overhydroxylation of Lys residues in type-I collagen in bone diseases. It has been long speculated that there is more than one type of LH; in turn, the tissue-specific collagen cross-linking pattern is, at least in part, presumably related to differential expression of the genes encoding LHs in the respective tissues.

In addition, we analyzed the gene expression of osteogenesis markers, such as *BGLAP* (osteocalcin), *COL1A1* (collagen 1A1), *MSX2* (homeobox gene MSX2), *SPARC* (osteonectin), and *VDR* (vitamin D receptor), by qRT-PCR. *BGLAP*, previously described as a late marker of developing osteoblasts, was observed significantly up-regulated in samples from BS and in healthy controls on the second day of differentiation. Mineralization could not be observed, though. *COL1A1* is known to be an early marker of osteoprogenitor cells [20]. Nonetheless, its highest level of expression was only reached on day 12. The homeobox gene *MSX2*, which is implicated in the osteoprogenitor cell function [21] and is an up-stream regulator of *RUNX2*, was up-regulated during the final phase of differentiation [22]. *SPARC* showed the maximum expression levels on the 12^th^ day of differentiation. Finally, *VDR* was maximally expressed on the 21^st^ day, concomitant with matrix mineralization. In all cases, the data obtained from BS patient showed that the expected expression profiles were quite similar to that of healthy donors, unlike the observed for OI patients. The gene expression of osteogenesis markers evaluated in this study suggested that, at least during osteogenic differentiation, BS-BMSCs are closer to healthy MSCs than to OI-BMSCs.

According to Stolzing *et. al* (2008), previous studies have addressed the effects of aging on MSCs, reporting age-related changes in MSCs, including loss of proliferation and differentiation potential, increases in senescent cell number and loss of in vivo bone formation potential [23]. In their work, they showed a progressive decline of specific ALP activity and chondrogenic differentiation in “aged MSC”, although adipogenic differentiation did not change significantly with age. However, It should also be emphasized that MSC from donors in the age range from 7 to 18 years were referred to as “young” MSC, for the age range 19–40 years “adult” MSC and for cells from over 40 years old donors were named “aged” by the authors. We cannot exclude the effect of age on the differentiation potential of MSCs, but we consider our conclusions are not found to be correlated with this specific factor, once all the OI, BS and normal subjects could be classified as “young” or “adult”.

Given the evidence that more than one locus contributes to the etiology of BS, accurate phenotype-genotype correlations will be important so as to understand the phenotypic heterogeneity of BS. Mutations in *PLOD2* and *FKBP10* can be a common etiology of BS, but future studies are needed to confirm the involvement of other genes in the heterogeneity of the phenotype.

Recent reports have detected *FKBP10* mutation in patients with BS and suggested that *FKBP10* deficiency might be the cause of Type-1 Bruck Syndrome. These results contribute to define BS as a highly heterogenic genetic disorder wherein several genes could be involved in the physiopathology.

## Conclusion

In summary, the present study is the first one that shows gene expression profiles of BMSCs from patients with BS and can be important to ascertain whether there are differences in clinical course that are determined by the nature of the underlying defect, especially considering the fact that there are different gene-expression data sets between BS and OI patients, at least for the genes analyzed in this study. These changes in gene expression may be related to phenotypic heterogeneity found in patients and their understanding may be important in determining new diagnostic strategies.

## Additional files

Additional file 1: Sequences of primers used for PCR, sequencing and qRT-PCR. (DOCX 18 kb) Additional file 2: Immunophenotype of Mesenchymal Stem Cells. Frequencies of cells positive for the indicated surface molecules in MSC. (DOCX 13 kb)

## Abbreviations

BGLAP: bone gamma-carboxyglutamate (gla) protein
BMSCs: bone marrow stem cells
BS: Bruck Syndrome
COL1A1: collagen, type I, alpha I
COL1A2: collagen, type I, alpha II
FKBP10: FK506 binding protein 10, 65 kDa
HC-FMRP-USP: Hospital das Clínicas da Faculdade de Medicina de Ribeirão Preto – Universidade de São Paulo
MNC: mononuclear cell fraction
MSC: mesenchymal stem cell
MSX2: Msh homeobox 2
No-RT: no reverse transcription control
NTC: no template control
OI: Osteogenesis Imperfecta
PCR: polymerase chain reaction
PLOD2: Procollagen-lysin, 2-oxoglutarate 5-dioxygenase 2
RT-PCR: real time polymerase chain reaction
SPARC: secreted protein, acidic, cysteine-rich (Osteonectin)
VDR: vitamin D (1,25- Dihydroxyvitamin D3) receptor
