# Supplementary material for: Osteoblastic differentiation of bone marrow mesenchymal stromal cells in Bruck Syndrome
Source: BMC Med Genet. 2016 May 4;17:38. doi: 10.1186/s12881-016-0301-7 (PMC4857408; doi:10.1186/s12881-016-0301-7)
Supplement: Additional file 1: — Sequences of primers used for PCR, sequencing and qRT-PCR. (DOCX 18 kb) [file 12881_2016_301_MOESM1_ESM.docx]

**Additional File 1 – Sequences of primers used for PCR, sequencing and qRT-PCR.**

| **Sequence** |
| --- |
| COL1A1-set1new-PF: CGGAGCAGACGGGAGTTTCT |
| COL1A1-set1new-PR: CCGAGTCTCCGGATCATCCA |
| COL1A1-set2new-PF: CGGGAAGTGAAAAATCCAAG |
| COL1A1-set2new-PR: GGAGAAGAAACAAGAGGCCA |
| COL1A1-set3new-PF: CTCCTGCCCTCGAATTTTGC |
| COL1A1-set3new-PR: GCACATGTCACAAACTGTGA |
| COL1A1-set4new-PF: CCAGGAAGTGCATGATGTCA |
| COL1A1-set4new-PR: GGTTAGAAGACAAGTCCCTG |
| COL1A1-set5new-PF: GGAGAGATGCTCAGAGATCT |
| COL1A1-set5new-PR: CCTTCCTCTGAGTATCGTTC |
| COL1A1-set6new-PF: CCAAAGAAGACTGAGACCTT |
| COL1A1-set6new-PR: GAGGTGCTTTTGGATGTCCA |
| COL1A1-set7new-PF: CCAAGGCTCTTTCTCAGATC |
| COL1A1-set7new-PR: GCTCCCATTGTCAGCCCCAA |
| COL1A1-set8new-PF: GGTTATGTTGGTCTGAACCC |
| COL1A1-set8new-PR: GTGGCACAGAGAAAGGAGTG |
| COL1A1-set9new-PF: CCCTTTGCCACTTTCTAACC |
| COL1A1-set9new-PR: GGCTCCTCTTCCTTTCTGGA |
| COL1A1-set10new-PF: CAGGAACCCCTGACACTGGA |
| COL1A1-set10new-PR: GCCTGATCCAGAACGCCTCA |
| COL1A1-set11new-PF: TGAGGCGTTCTGGATCAGGC |
| COL1A1-set11new-PR: CCAGGACTCCTTCAAGTCTC |
| COL1A1-set12new-PF: CCACTCAGAGTAAATGAGAG |
| COL1A1-set12new-PR: CCTCTAGTTGATGGCTGTCT |
| COL1A1-set13new-PF: GGAAGGACCGTGCTTTCCAG |
| COL1A1-set13new-PR: ATCTCCATGGCTTTGGTCAT |
| COL1A1-set14new-PF: GGAAACAAGCCTGGGAGATA |
| COL1A1-set14new-PR: CCAGAGAGAAGGAGAGATGC |
| COL1A1-set15new-PF: GCAGAGGGCCTCTCAGGAAA |
| COL1A1-set15new-PR: GGAGTCAGATTGGAGAGATG |
| COL1A1-set16new-PF: GTACAGAAGACCTGTTAAGA |
| COL1A1-set16new-PR: GAGAGCACAGAGGCATCAAG |
| COL1A1-set17new-PF: TGAGTGGCTTGGCCCTCTGT |
| COL1A1-set17new-PR: AGGTGCCAGAGAGCAGCACA |
| COL1A1-set18new-PF: GTGCCAGCTCAGATCTCTGC |
| COL1A1-set18new-PR: AGAGAGAGAGAAGTGAGAGT |
| COL1A1-set19new-PF: CCTGGTGAATCTGGACGTGA |
| COL1A1-set19new-PR: CCTGACATCTTGCAGGATCT |
| COL1A1-set20new-PF: TTGGGAGAGATGGCCACAGT |
| COL1A1-set20new-PR: CCATGCCCCTTCATTATTCT |
| COL1A1-set21new-PF: AGAATAATGAAGGGGCATGG |
| COL1A1-set21new-PR: GGATTACCGGCATCCAAGTG |
| COL1A1-set22new-PF: GGCAAAGATGGACTCAACGG |
| COL1A1-set22new-PR: TTGGGGTCAATCCAGTACTC |
| COL1A1-set23new-PF: GCCACTCTGACTGGAAGAGT |
| COL1A1-set23new-PR: CCCAATGCACCGTTATATCG |
| COL1A1-set24new-PF: CGATATAACGGTGCATTGGG |
| COL1A1-set24new-PR: GGAGGTCTTGGTGGTTTTGT |
| COL1A1-set25new-PF: GCTTCACCTACAGCGTCACT |
| COL1A1-set25new-PR: GGAGAAAGGAGCAGAAAGGG |
| COL1A1-set26new-PF: CCAAAAGTGCATTCAACCTT |
| COL1A1-set26new-PR: CCATCACATAGATGTAGCAC |
| COL1A1-set27new-PF: GGAGAGACTGTTCTGTTCCT |
| COL1A1-set27new-PR: GGGTCATTTCCACATGCTTT |

| **Sequence** |
| --- |
| COL1A2-exon1-PF: CCTACAAGTGGCCTACAGGG  COL1A2-exon1-PF: CCTCCCATCTAACCTCTCTA |
| COL1A2-exon2-PF: GACCTGCATAATTTCTAGGT  COL1A2-exon2-PR: TTCATAGAAGCTGATCCTAA |
| COL1A2-exon3-PF: TTAGGATCAGCTTCTATGAA  COL1A2-exon3-PR: CACCAGTTTGTATCACATAA |
| COL1A2-exon4-PF: GCAGCTTCCAATCCTCCAGC  COL1A2-exon4-PR: GGACTGTGGTGGTAGGTAGA |
| COL1A2-exon5-PF: CCCTGTGATATCTTAAGAGT  COL1A2-exon5-PR: CAGTGCACACAAAGACCAGT |
| COL1A2-exon6-PF: GAGGTGTCGGCCAAGTTTTT  COL1A2-exon6-PR: CCCAAGTTATGGTTACTATG |
| COL1A2-exons7a9-PF: GGAATCAAACCACAACAATG  COL1A2-exons7a9-PR: CCATACATTAAGAGTTCTGT |
| COL1A2-exon10-PF: ACAGAACTCTTAATGTATGG  COL1A2-exon10-PR: TCTAAGCACAGAGTGACAAA |
| COL1A2-exon11-PF: TTTGTCACTCTGTGCTTAGA  COL1A2-exon11-PR: CCCTAGATAGGTCACTTAAC |
| COL1A2-exon12-PF: GCTGGGACCTGGAACACTGGACTTC  COL1A2-exon12-PR: TGGAGGTCATGGGGAATTTCAATCA |
| COL1A2-exons13a15-PF: CTGTGTGTCTGGCATAATTG  COL1A2-exons13a15-PR: AATGAAGAAGACAGCACCCA |
| COL1A2-exon16-PF: GTGTCATGCCACTGTAAGCA  COL1A2-exon16-PR: CTCTCTGTGGTTGACTCTGG |
| COL1A2-exon17-PF: CAGTAGCCAAGATGGCAGAATC  COL1A2-exon17-PR: CCAGTAAGGCCGTTTGCTCCAG |
| COL1A2-exon18-PF: CGTTGGACCTCCTGTAAGTAG  COL1A2-exon18-PR: AAAATGCAGTGTGGTCCATTAGG |
| COL1A2-exon19-PF: TAATGTGTGCTGCCTCTACAGC  COL1A2-exon19-PR: CATATAGCAGACGGGAGTGTAC |
| COL1A2-exon20-PF: CTTGAGCTTCTCTTTACCTTGAC  COL1A2-exon20-PR: CACCACTGGGACCAGGAGGAC |
| COL1A2-exon21-PF: CGTAAGTAGCTCTATCATCAC |
| COL1A2-exon21-PR: AAGGCAGATGGAAAGCAGATG |
| COL1A2-exons22a23-PF: GCAGGATGCTCATCTATGAA  COL1A2-exons22a23-PR: CTGTCAGCAAGACTACTAAC |
| COL1A2-exon24-PF: AAAAAGTCGGGGGAAAAGGTGCCTT  COL1A2-exon24-PR: TCTCCCCTGCTCTGCTTTCAGTCCT |
| COL1A2-exon25-PR: TCCCTGAGACTGGACTGATT |
| COL1A2-exon26-PF: CCACAGACTAGGGATCTCAA  COL1A2-exon26-PR: GCTACTACATATTCATACCC |
| COL1A2-exons27a28-PF: CGTGGGAACCCACAATGAGT  COL1A2-exons27a28-PR: CCAAATATCAACATGAGCAC |
| COL1A2-exon29-PF: GAGCTGTAAATCACCATACCGTAC  COL1A2-exon29-PR: TGGCTCATTCTCTCCATCAGCAC |
| COL1A2-exon30-PF: GCACTCATGTAGATACTGCC  COL1A2-exon30-PR: GGCTTTGAACATCAACACAC |
| COL1A2-exon31-PF: CTAGTGGAGAGATTAGGAAC  COL1A2-exon31-PR: CCACTGGAATCGGATTGCTG |
| COL1A2-exon32-PF: GCAGGCAAGAAGCCTGTCTA  COL1A2-exon32-PR: CCTCATGTATTACTCAACAC |
| COL1A2-exon33-PF: GAATGGTAAGGAATCGAGACATTGC  COL1A2-exon33-PR: AATTTGGAAAATTCTCAATTCAACATAAAAAAAAATCCAAGTACGAAG |
| COL1A2-exon34-PF: CTCCTTCTGAGAGTGGCTTC  COL1A2-exon34-PR: CCTGCTGCTCTATCACAATA |
| COL1A2-exons35a37-PF: GTCAGTTATCTCTTCCAAGG  COL1A2-exons35a37-PR: CCTGTTGCATAGCAGGCACT |
| COL1A2-exon38-PF: GGTGGTAATATTGAAGAACA  COL1A2-exon38-PR: GCTGATAGCAACATACACTG |
| COL1A2-exon39-PF: GGTCTATTCCTGGTCACATG  COL1A2-exon39-PR: ACTTCAGACCAGGAGAGTAA |
| COL1A2-exon40-PF: CCAAATGGCCAGGGTATTAT  COL1A2-exon40-PR: GGCTCAACTGAGCTCTACTT |
| COL1A2-exon41-PF: GCCAAGATGTAAACTCACCG  COL1A2-exon41-PR: GCTGTGTCTTTATAGTGTGT |
| COL1A2-exon42-PF: CCTTCTTCCTTCAAACTAGA  COL1A2-exon42-PR: CCATTCTTTGGCCTAAGCAA |
| COL1A2-exons43a45-PF: GTGATGAAGACAGAGTAGCT  COL1A2-exons43a45-PR: CAGATGTTTTGGACTGATTC |
| COL1A2-exon46-PF: GTGAGAGCCTAGCTAAACCA  COL1A2-exon46-PR: GCCAGAGAATGGGAAATGGA |
| COL1A2-exons47a48-PF: GAGCCCCACTTTACATTTTC  COL1A2-exons47a48-PR: GAGGATATATTGAAATGGGG |
| COL1A2-exon49-PF: CTGATGAGAACATGCTTCCG  COL1A2-exon49-PR: CATATTTAAGAGGAAGAGGG |
| COL1A2-exon50-PF: CCCTCTTCCTCTTAAATATG  COL1A2-exon50-PR: CCAATCAATCCATCTTCTAA |
| COL1A2-exon51-PF: CCCTTTTCCTAAGCTTGGAT  COL1A2-exon51-PR: GTTAGTTCTCTCATTCTTCT |
| COL1A2-exon52.1-PF: GGGACAGACATCTTCAGAAT  COL1A2-exon52.1-PR: GGAAAGTGTTTTGAGGTAGT |
| COL1A2-exon52.2-PF: CAACACTCTTACACCTGTTA |
| **Sequence** |
| PLOD2-exon1-PF: CCGACGACCTCACTCAGCTG  PLOD2-exon1-PF: AGGGCTGGTGGATGAATGAA |
| PLOD2-exon2-PF: CGACAGCTCTTTGTTTAGTC  PLOD2-exon2-PR: GAGGATTACAGATTGTACTG |
| PLOD2-exon3-PF: CCATGAAACATTAGAAGAGT  PLOD2-exon3-PR: GCCCAACCATATTTTAATAC |
| PLOD2-exon4-PF: GTTCTGTGTAAATAGTTGGC  PLOD2-exon4-PR: CAACTCACATAATACATGGG |
| PLOD2-exon5-PF: CATGGTGAGCTGTGAAATTC  PLOD2-exon5-PR: CAGTTTACCATTTGCCATAC |
| PLOD2-exon6-PF: GAGTTGCAACTATCGCAGTT  PLOD2-exon6-PR: ACTGTCGACCTTAGTCACAG |
| PLOD2-exons7-PF: GATGATACACACACATACAC  PLOD2-exons7-PR: TAGATGACATTCCACATATC |
| PLOD2-exon8-PF: GTGTGCACCTACAGATGATA  PLOD2-exon8-PR: CCCCATTATCTATCATAAAA |
| PLOD2-exon9-PF: GGAGCAATTCAAATTTAGCA  PLOD2-exon9-PR: CCTCCACTGAACTTAACCCA |
| PLOD2-exon10-PF: CCGTGTTTAATATGGTTATT  PLOD2-exon10-PR: GAGACACCCAAATTTGGCAT |
| PLOD2-exons11-PF: GCTAGAGAACCATATTTTAC  PLOD2-exons11-PR: ACTACAGTATGTTCACATC |
| PLOD2-exon12-PF: CTACAGGTTTGTTGAATGAG  PLOD2-exon12-PR: GGGTTCTCAAATTCCCATTA |
| PLOD2-exon13-PF: GGCAGTGGTTTATCTCCTAA  PLOD2-exon13-PR: GGCTAGTACTTACAAATTAG |
| PLOD2-exon14-PF: GAGCTGGAGATGTTTCTTTG  PLOD2-exon14-PR: CCAAGCTAAATGCAAATAAG |
| PLOD2-exon15-PF: GTTTTGTGTGGTGTCTGAAT  PLOD2-exon15-PR: GATGTGCATTTCACCTTATG |
| PLOD2-exon16-PF: GGCAAAGATTAGGCTATATA  PLOD2-exon16-PR: ACTTTCACATCTTCTGTGAA |
| PLOD2-on17a19-PR: GCCACAACTTCAAAGACGTGT |

| **Primer** |
| --- |
| FKBP10-set1-PF: GGGACTAGAATTCGAAGTGC  FKBP10-set1-PR: GGAACATGGAGCCTGGAGTT |
| FKBP10-set2-PF: GGAAAGAGGACAGGAAGAGG  FKBP10-set2-PR: GAGTATTGGGATGCAGAGCT |
| FKBP10-set3-PF: GGAGGTGAGAAGTGTGTGTG  FKBP10-set3-PR: GCCACTCTTCATACTGCGTG |
| FKBP10-set4-PF: GTGGCATCTCTGTCCCTGGT  FKBP10-set4-PR: CCAGAGCCGACGTAGGTGTC |
| FKBP10-set5-PF: GCGACTTTGTCCGCTACCAC  FKBP10-set5-PR: CCCTCGTGCAGTATACAACC |
| FKBP10-set6-PF: GGAGCAAGAAGCAGGGCTGC  FKBP10-set6-PR: GCTGCATGCATGTTTTTGTG |
| FKBP10-set7-PF: GTGAGTGGAAAAGGGCTTTC  FKBP10-set7-PR: GCAGAGAATTTCATCTCCTC |
| FKBP10-set8-PF: GAGCCCTCGAGGCCACACTT  FKBP10-set8-PR: GGCACGGTGGCAGGTGCCTG |
| FKBP10-set9-PF: GGAGTCAGGAATGCCTTCAG  FKBP10-set9-PR: GGATAGTCTGTGACTGGTCT |
| FKBP10-set10-PF: AGACCAGTCACAGACTATCC  FKBP10-set10-PR: GGGATGGAAGAGAAACACGG |
| FKBP10-set11-PF: TGAGGTCCAGGAGCCAACTAA  FKBP10-set11-PR: CCCAATCTCTGGGTCTAAAG |

| **Sequence** |
| --- |
| MSX2-RT-PF: TTACCACATCCCAGCTCCTC  MSX2-RT-PR: CCTGGGTCTCTGTGAGGTTC |
| VDR-RT-PF: CCAACCCATCAGAAGGAGAA  VDR-RT-PR: TATCCAAGGCTGAGGTGGAG |
| COL1A1-RT-PF: CCTGGATGCCATCAAAGTCT  COL1A1-RT-PR: TCTTGTCCTTGGGGTTCTTG |
| SPARC-RT-PF: CTGGGCTCTGCCTTAAACAC  SPARC-RT-PR: GCCAAGACCCTGAAATGAAA |
| GAPDH-SYBR-PF: ACCCACTCCTCCACCTTTGA  GAPDH-SYBR-PR: CTGTTGCTGTAGCCAAATTCGT |
|  |
|  |
