# Supplementary material for: Osteoblastic differentiation of bone marrow mesenchymal stromal cells in Bruck Syndrome
Source: BMC Med Genet. 2016 May 4;17:38. doi: 10.1186/s12881-016-0301-7 (PMC4857408; doi:10.1186/s12881-016-0301-7)
Supplement: Additional file 2: — Immunophenotype of Mesenchymal Stem Cells. Frequencies of cells positive for the indicated surface molecules in MSC. (DOCX 13 kb) [file 12881_2016_301_MOESM2_ESM.docx]

**Additional File 2** – Immunophenotype of Mesenchymal Stem Cells. Frequencies of cells positive for the indicated surface molecules in MSC.

|  | **CD45** | **CD14** | **CD34** | **CD31** | **CD29** | **CD13** | **CD90** | **CD73** | **CD105** |
| --- | --- | --- | --- | --- | --- | --- | --- | --- | --- |
|  |  |  |  |  |  |  |  |  |  |
| **NBM1** | 0,16 | 0,62 | 0 | 1,00 | 97,15 | 98,65 | 99,10 | 96,44 | 97,38 |
| **NBM2** | 0,20 | 0,69 | 0,49 | 1,08 | 96,28 | 98,83 | 97,17 | 95,45 | 97,35 |
|  |  |  |  |  |  |  |  |  |  |
| **OI-1** | 0,18 | 1,07 | 0,16 | 0,33 | 94,8 | 98,56 | 99,07 | 95,99 | 96,24 |
| **OI-2** | 0,01 | 1,43 | 2,34 | 4,43 | 83,85 | 95,88 | 91,43 | 78,54 | 92,32 |
| **OI-3** | 1,74 | 3,36 | 0 | 0,38 | 93,32 | 96,36 | 97,33 | 94,24 | 94,51 |
| **OI-4** | 0,29 | 0,30 | 2,74 | 0,34 | 96,20 | 96,41 | 99,11 | 96,23 | 95,38 |
| **BS** | 0 | 0,23 | 0,12 | 0,10 | 96,33 | 94,31 | 97,99 | 92,65 | 91,44 |

Legend: OI1-4: Osteogenesis Imperfecta patients; BS: Bruck Syndrome patient; NBM1-2: normal bone marrow donors.
